# Muscle activation patterns and muscle synergies reflect different modes of coordination during upper extremity movement

**DOI:** 10.3389/fnhum.2022.912440

**Published:** 2023-01-18

**Authors:** Xiaoling Chen, Xiaojiao Dong, Yange Feng, Yuntao Jiao, Jian Yu, Yan Song, Xinxin Li, Lijie Zhang, Peiguo Hou, Ping Xie

**Affiliations:** ^1^Key Laboratory of Measurement Technology and Instrumentation of Hebei Province, School of Electrical Engineering, Yanshan University, Qinhuangdao, Hebei, China; ^2^Key Laboratory of Intelligent Rehabilitation and Neuromodulation of Hebei Province, School of Electrical Engineering, Yanshan University, Qinhuangdao, Hebei, China; ^3^School of Mechanical Engineering, Yanshan University, Qinhuangdao, Hebei, China

**Keywords:** muscle synergies, muscle activation patterns, surface electromyography, motor control, non-negative matrix factorization

## Abstract

A core issue in motor control is how the central nervous system generates and selects the muscle activation patterns necessary to achieve a variety of behaviors and movements. Extensive studies have verified that it is the foundation to induce a complex movement by the modular combinations of several muscles with a synergetic relationship. However, a few studies focus on the synergetic similarity and dissimilarity among different types of movements, especially for the upper extremity movements. In this study, we introduced the non-negative matrix factorization (NMF) method to explore the muscle activation patterns and synergy structure under 6 types of movements, involving the hand open (HO), hand close (HC), wrist flexion (WF), wrist extension (WE), supination (SU), and pronation (PR). For this, we enrolled 10 healthy subjects to record the electromyography signal for NMF calculation. The results showed a highly modular similarity of the muscle synergy among subjects under the same movement. Furthermore, Spearman’s correlation analysis indicated significant similarities among HO-WE, HO-SU, and WE-SU (*p* < 0.001). Additionally, we also found shared synergy and special synergy in activation patterns among different movements. This study confirmed the theory of modular structure in the central nervous system, which yields a stable synergetic pattern under the same movement. Our findings on muscle synergy will be of great significance to motor control and even to clinical assessment techniques.

## Introduction

Human movement is a highly complex activity produced by neuromuscular activation and biomechanical output (Gottlieb, 1998). It is a common assumption that the central nervous system (CNS) with a modular structure can simplify motor tasks to low-dimensional modules by linear combinations of muscle synergy (Cheung et al., 2012; Bizzi and Cheung, 2013; d’Avella and Lacquaniti, 2013), which refers to several muscles participating in a movement in a fixed combination (Flash and Bizzi, 2016; Liang et al., 2021). Additionally, some studies pointed out that the CNS is endowed with a set of intrinsically representative synergetic modules, and it can dominate some of the modules as a combination to involve different movements (Israely et al., 2018; Gueugnon et al., 2019; Cheung et al., 2020; Jonsdottir et al., 2020). However, it is still unclear how to choose the muscle activation pattern, and organize and coordinate muscles to mobilize different behaviors and movements.

Over the last few years, extensive studies have sought methodologies to elucidate muscle synergy. The present studies mainly depended on the theories of dimension reduction and blind source separation, such as independent component analysis (ICA), principle component analysis (PCA), second-order blind identification (SOBI), and non-negative matrix factorization (NMF). Here, with respect to both ICA and PCA, two types of blind source separation can reveal several patterns of muscle synergy but have limitations in the specific assumptions in the extracted muscle synergy (orthogonality for PCA and statistical independence for ICA) and the quite highly mean communality of the data (Ivanenko et al., 2004; Weiss and Flanders, 2004; Esmaeili and Maleki, 2019). Later, some studies tried to use the SOBI method for muscle synergy estimation, but it is the best algorithm with four channels (no dimension reduction) and is not suitable for this study (Belouchrani et al., 1997; Ebied et al., 2018). Compared with the above methods, the NMF method has its advantages in extracting the synergetic module by decomposing the EMG matrix into several low-dimensional spaces and time-dependent variables, which is widely applied in muscle synergy (Amundsen Huffmaster et al., 2018; Bahadur et al., 2019; Matsuura et al., 2020). Recently, many studies have explored muscle synergy and its similarities between upper-and lower-limb movements (Rabbi et al., 2020; Chen et al., 2021). Additionally, the NMF method can be used to decompose signals into the non-negative elements in the matrix and has better robustness. Therefore, we applied the NMF algorithm to extract muscle synergy under different upper extremity movements.

Recently, several studies also have set out to investigate the synergetic similarity and dissimilarity among different types of movements. A previous study pointed out that the synergetic similarity named shared synergy, meant the synergetic module participated in more than one movement, while the synergetic dissimilarity, called special synergy, was just involved in the specific movements without involvement in other tasks (Russo et al., 2014). The shared synergy and special synergy have become a biomarker to explore the muscle activation pattern among different movements (Torres-Oviedo and Ting, 2010; Nazifi et al., 2017). For example, Roh et al. (2011) found some muscle synergies shared in jumping, swimming, kicking, and walking in frogs. Boccia et al. (2018) found a shared synergy between Nordic walking and traditional walking. Additionally, Allen et al. (2017) found that the shared synergy was highly consistent during walking, and the special synergy also had a lesser consistency in muscle synergy from mild to moderate for patients with Parkinson’s disease. The above studies showed that the shared synergy and specific synergy can represent consistent motor modules that map intention to movement and reflect the synergetic physiological mechanism (Singh and Latash, 2011; Yun et al., 2015; Afzal et al., 2017). However, similar studies mostly focus on the lower limb movement, and few reports focus on the upper extremity. It is worthy of deep consideration to explore the muscle activation pattern and the combinations of muscle synergy (shared synergy or special synergy) to mobilize upper extremity movement.

Mostly, the main contribution of this study is to explore the muscle activation patterns and muscle synergy among different modes of coordination during upper extremity movement. For this, we introduced the NMF method to the electromyography (EMG) signals of upper extremity movement from 10 subjects under 6 types of upper extremity movements. Consequently, we used Spearman’s correlation analysis to quantify the similarity of different movements. We analyzed the synergy similarity within the same movement and among different movements. Furthermore, we compared the cooperative structure with the muscle activation pattern and extracted the special synergy and shared synergy. The present study explores the cooperative mechanism of the upper extremity movement by the nervous system.

## Materials and methods

### Subjects

Ten healthy right-handed subjects (7 men and 3 women; mean age, 24.6 ± 1.51 years; range, 23–28 years) were enrolled in this study. All participants had no history of upper-limb motor dysfunction or joint injury. They participated according to the declaration of Helsinki and gained the consent and approval of the Ethical Review Board of Yanshan University. All participants had not exercised vigorously within 24 h before the test to remove the influence of fatigue.

### Experimental paradigm

**FIGURE 1:**
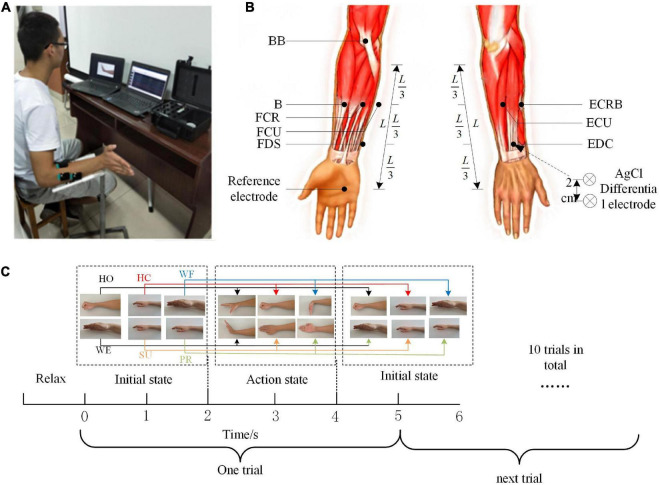
Experiment program. **(A)** Recording of 8-channel electromyography (EMG). **(B)** Electrode positions for the experiment on the acquisition of EMG signals. **(C)** The flow of the experimental task.

Each subject was asked to complete six types of upper extremity movements, including hand open (HO), hand close (HC), wrist flexion (WF), wrist extension (WE), supination (SU), and pronation (PR), as shown in Figure 1C. All subjects were required to sit on the test chair, with the upper arm retracted close to the ribs, the elbow joint appressed to the body, while the elbow bent 90 degrees, and the forearm forward (Figure 1A). To avoid interference by other muscles, the subjects are not permitted to swing their shoulders during the test. The standard of movement during collecting was to reach the most standard maximum position in the experimental process according to the clues. The tasks were given in Figure 1C. In the entire task, there were 4 sessions with a 60-s break between each session, and each session included 50 s and contained 10 trials. The subjects had a 5-min break after each set of movements to relax the arm muscles and avoid muscle fatigue.

### EMG data recording and preprocessing

In this study, the EMG signal was recorded by an 8-channel Trigno*™* Wireless EMG system (Delsys Inc., Natick, MA, USA). The system’s built-in bandpass filter was set at 5–500 Hz, and all recorded EMG data were sampled at 1,000 Hz per channel. Before the electrode application, the skin surface was cleaned with alcohol. We simultaneously collected the EMG data from eight muscles in the right upper limb, involving flexor digitorum superficialis (FDS), extensor digitorum communis (EDC), brachioradialis (B), radial carpi flexor muscles (flexor carpi radialis, FCR), flexor carpi ulnaris (FCU), extensor carpi radialis brevis (ECRB), extensor carpi ulnaris (ECU), and biceps (biceps brachii, BB). The position of each electrode was shown in Figure 1B.

To obtain effective EMG signal characteristics, we needed to preprocess the original signal. First, we used the fourth-order Butterworth high-pass filter (cutoff frequency of 40 Hz) to filter the signal and then removed the means and rectification (Myers et al., 2003). Then, we used the fourth-order Butterworth low-pass filter (cutoff frequency of 4 Hz) to extract the signal envelope and introduced the maximum normalization method (Esmaeili and Maleki, 2019; Korak et al., 2020) to normalize each channel EMG data for every trial according to the maximum amplitude value in each channel. We preprocessed all signals for each movement according to the above methods and built a data matrix of *L*×*K* (*L* was the number of muscles, *K* was the number of data points), which was set as the muscle activation matrix *M*.

### Muscle synergy extraction method

#### Muscle activation model based on synergy

Previous studies have pointed that the activation model of muscle *M* can be regarded as a linear combination of the muscle synergy recruitment coefficient *C*_i_(*t*) (activation coefficient) and the muscle synergy vector of recruitment *W*_i_ (Wenger et al., 2016). The activation model of muscle can be expressed as follows:


(1) $$M\;\left(t\right)=\sum_{i=1}^{N}W_{i}\,C_{i}\,\left(t\right)$$

where *i* = 1,2,…,*N* is the number of muscle synergies, and *M*(*t*) is the *M*-dimensional vector of the activation degree at point *t*. *C*_i_(*t*) is the activation coefficient, which indicates how the *i* muscle synergy is modulated at the moment *t* and reflects the contribution of each muscle synergy to muscle excitation. *W*_i_ is the muscle synergy vector, which represents the relative weight of each muscle in the *i* muscle synergy. We can use formula (1) to reverse the simulation reconstruction by setting the activation coefficient *C*_i_(*t*) and the muscle synergy vector *W*_i_.

#### Non-negative matrix factorization

To analyze the essence of muscle synergy in the movement, it is necessary to select an effective method to decompose the EMG signal. In this study, we introduced the NMF algorithm (Dwivedi and Shibata, 2019) to extract muscle synergy. The NMF method can obtain the mapping matrix by projecting the high-dimensional data into the low-dimensional subspace and decomposing the high-dimensional matrix into the multiplication of two low-dimensional matrices. The NMF algorithm can be approximated as the following structure:


(2) $$V_{i\,\mu }\approx {\left(W\,C\right)}_{i\,\mu }=\sum_{\alpha =1}^{r}W_{i\,\alpha }\,C_{\alpha \,\mu }$$

where *W*_iα_ denotes the base matrix, and *C*_αμ_ is the coefficient matrix. The column vector of the matrix *V*_*i*μ_ can be interpreted as the weighted sum of all the column vectors *W*_iα_, and the weight coefficients are the elements in the corresponding column vector *C*_αμ_. The reconstructed matrix $V_{i\,\mu }^{\prime }$ is obtained by multiplying the matrices *W*_iα_ and *C*_αμ_, which can be obtained from the matrix *V*_*i*μ_. The consistency of the matrix is quantified by calculating the sum of the squared errors ${\left(V_{i\,\mu }-V_{i\,\mu }^{\prime }\right)}^{2}$, and the iterative optimization is continued until the sum of the squared errors of the base matrix and coefficient matrix is minimum.

This study uses the above muscle activation model to decompose the muscle activation pattern matrix *M* of all movement cycles under different movements of each subject. The muscle activation model matrix is decomposed to obtain two matrices of muscle coordination *W* and activation scale coefficient *C*. Among them, *M* has *L* rows and *K* columns (*K* is the number of sampling points), *W* is a matrix of *L* rows and *N* columns (*N* is the number of coordination), and *C* is a matrix of *N* rows and *K* columns. The matrix obtained after decomposition is quantified for consistency to get the optimal target matrix *W* and *C*.

### Determination of the minimum synergy number

To determine the number of columns of the matrix *W* in the above decomposition, namely, the minimum synergy number, we introduced the variability accounted for VAF calculation method, which is defined as follows:


(3) $$V\,A\,F=1-\frac{R\,S\,S}{T\,S\,S}=1-\frac{\sum {\;(M_{E\,M\,G}-M_{E\,M\,G}^{\prime })}^{2}}{\sum M_{E\,M\,G}^{2}}$$

where RSS and TSS are the residual and total sum of squares, respectively. *M*_*EMG*_ is the original muscle activation model matrix, and $M_{E\,M\,G}^{\prime }$ is the data matrix reconstructed by the NMF. The square in the molecule is calculated as the square of each element in the new matrix, which is the difference between the two matrices.

The VAF of the muscle activation models under different synergy numbers can be calculated by the formula (3). To determine the number of decomposition columns of the matrix *W*, the *N* is considered as the minimum synergy number, when the mean of VAF is greater than 90% under a certain value of *N* and if one more synergy number is added, and the VAF increment is less than 5% (Santuz et al., 2017).

### Statistical analysis

In our study, we used the two-way ANOVA to analyze the difference between the VAF of each movement under different numbers of synergy and then used the LSD test for multiple comparisons. Additionally, we used Spearman’s correlation analysis to calculate the similarity coefficient *r* and the significance *p*-value of the average muscle synergy under different movements and make comparisons among each paired movement. We set up the thresholds for *r* referring to existing studies and the control mechanism of upper limb movements (Torres-Oviedo and Ting, 2010; Nazifi et al., 2017). When the correlation reaches *r* > 0.500 and *p* < 0.05, we believe that there is a statistical similarity between different synergetic modules to extract the shared synergetic module for each movement; when the correlation reaches *r* < 0.400 and *p* > 0.05, it is considered no statistical similarity among synergetic modules; and the synergetic module is judged to be a special synergetic module, when the correlation reaches 0.400 < *r* < 0.500, and it is viewed that there may be errors caused by limited data collection or individual differences in the experiment.

## Results

### Modular numbers and structure for muscle synergy

**FIGURE 2:**
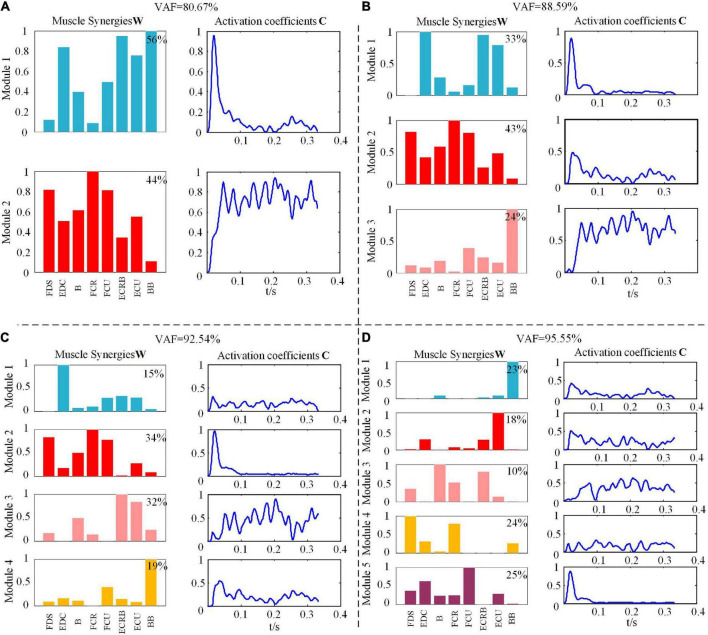
Muscle synergies matrix and synergy activation coefficient under different module amounts during wrist flexion (WF) movement. **(A)** The value of variability accounted for (VAF) is 80.67%. **(B)** The value of VAF is 88.59%. **(C)** The value of VAF is 92.54%. **(D)** The value of VAF is 95.55%.

Figure 2 shows the NMF decomposition results with various conditions of the VAF values for one subject under the WF movement. Here, we could find that the VAF value was 88.59% as the synergy number was 3, and the VAF value got to 92.54% along with the number increasing to 4. When the synergetic number added one more, the VAF value became 95.55% with less than 5% increments.

**FIGURE 3:**
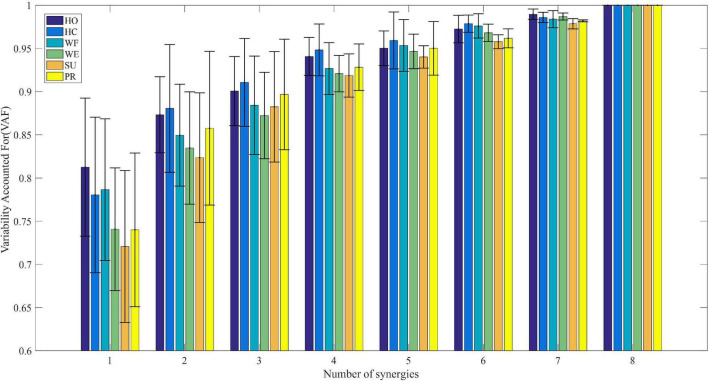
The relationship between average variability accounted for (VAF) and module amount under different upper limb movements.

To further exhibit similar trends for all subjects, we calculated the mean VAF values for all subjects under each movement with the modular numbers of muscle synergy increasing from 1 to 8. Figure 3 shows that the mean VAF values increased as the number of synergies increased, although the period of a relative slowdown of growth continued from 4 to 8. We found that when the number of synergies ranged from 1 to 4, the VAF values of HO and HC movements were higher than the other four movements (*p* < 0.01). However, there was no significant difference in the VAF values of the six movements, when the number of synergies was high (*p* < 0.05). It could be seen that when the number of synergies was 4, the mean VAF values of the six movements were greater than 90%, and the increase of VAF was less than 5% after adding one more synergy, so the number of synergies of the six movements was determined to be 4. According to the principle in Section “2.5 Determination of the minimum synergy number,” we considered the muscle synergy matrix with four modules as the effective synergy structure.

### Synergetic similarity analysis

#### Synergetic similarity within the same movement

To analyze the similarity of muscle synergy among subjects under the same movement, we applied Spearman’s correlation analysis to compare similar pairs of synergetic modules. Table 1 listed the results for all subjects during HO movement, and showed that each subject yielded a basically similar synergy. We also drew the same conclusion in other movements, which indicated a stable muscle synergetic pattern among subjects under the same movement.

**TABLE 1:** The correlation coefficients _*r*_ of muscle synergies and similar pairs among subjects during hand open (HO) movement.

	Subject 1		Subject 2		Subject 3		Subject 4		Subject 5		Subject 6		Subject 7		Subject 8		Subject 9		Subject 10	
	_*r*_	pairs	_*r*_	pairs	_*r*_	pairs	_*r*_	pairs	_*r*_	pairs	_*r*_	pairs	_*r*_	pairs	_*r*_	pairs	_*r*_	pairs	_*r*_	pairs
*Subject*1	1.000**	4	-		-		-		-		-		-		-		-		-	
*Subject*2	0.682**	4	1.000**	4	-		-		-		-		-		-		-		-	
*Subject*3	0.626**	4	0.549	3	1.000**	4	-		-		-		-		-		-		-	
*Subject*4	0.745**	4	0.635**	4	0.695**	4	1.000**	4	-		-		-		-		-		-	
*Subject*5	0.496	3	0.595**	4	0.519	3	0.500	3	1.000**	4	-		-		-		-		-	
*Subject*6	0.657**	4	0.585**	4	0.523	3	0.624**	4	0.635**	4	1.000**	4	-		-		-		-	
*Subject*7	0.518	3	0.619**	4	0.796**	4	0.598**	4	0.640**	4	0.481	3	1.000**	4	-		-		-	
*Subject*8	0.551*	4	0.654**	4	0.722**	4	0.630**	4	0.639**	4	0.608**	4	0.628**	4	1.000**	4	-		-	
*Subject*9	0.653**	4	0.660**	4	0.720**	4	0.475	3	0.515	3	0.569*	4	0.559*	4	0.623**	4	1.000**	4	-	
*Subject*10	0.695**	4	0.595**	4	0.649**	4	0.709**	4	0.523	3	0.570*	4	0.675**	4	0.572*	4	0.636**	4	1.000**	4

*P < 0.05, **_P_ < 0.001, -Omit.

#### Averaged muscle synergy under different movements

**FIGURE 4:**
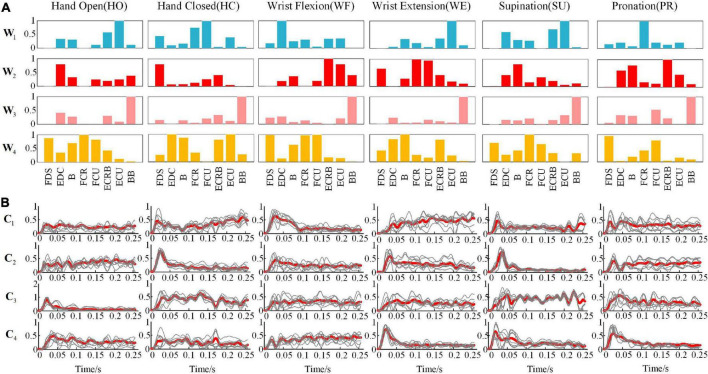
Under different movements **(A)** the representative muscle synergies matrixes and **(B)** average synergy activation coefficients (the thin line is the activation scale coefficient of 10 subjects, and the thick line is the superimposed average).

Considering the similarity and robustness of the muscle synergy for all subjects, Figure 4 shows the averaged results of the synergetic modules and activation coefficients extracted from each movement. Hereby, the variable $\bar{W_{i}}\left(i=1,2,3,4\right)$ represented the four modules, and $\bar{C_{i}}\left(i=1,2,3,4\right)$ was the corresponding activation coefficients. To elaborate on the dominant muscle combinations in each $\bar{W_{i}}$ module for all movements, we calculated them and listed them in Table 2. For example, when we performed the HO movement, the main components were ECU and ECRB in the module $\bar{W_{1}}$; EDC in the module $\bar{W_{2}}$; BB in the module $\bar{W_{3}}$; and FCR, FDS, and FCU in the module $\bar{W_{4}}$, respectively. All modules coordinated with each other to achieve the HO movement, and there was a similar phenomenon in other movements. Although each subject showed a different trend of the activation coefficients for each movement, all subjects presented the same trend for a certain fixed movement.

**TABLE 2:** The analysis of major activated muscle groups in different modules under different actions.

Module	Action name					
	HO	HC	WF	WE	SU	PR
$\bar{W_{1}}$	ECU, etc. Carpi radiali is extend	FCU, FCR, etc. Carpi radiali is adduction	EDC is active Fingers bend	ECU, etc. The active muscle of the wrist extension	ECU, ECR and other extensor muscle group	FCR is the active muscle
$\bar{W_{2}}$	EDC is the active muscle	FDS is the active muscle	ECRB, ECU and other extensor sets Main antagonistic muscles of wrist flexion	FCU, FCR, etc. Main antagonistic muscle groups of wrist extension	B is the active muscle Keep the wrist flexed	B, ECRB, etc. Auxiliary wrist inward turning
$\bar{W_{3}}$	BB maintain smooth upper limbs	BB maintain smooth upper limbs	BB maintain smooth upper limbs	BB maintain smooth upper limbs	BB maintain smooth upper limbs	BB maintain smooth upper limbs
$\bar{W_{4}}$	Antagonistic flexor collection	Antagonism extensor collection	FCR, FCU is the active muscle Wrist adduction	Flexor muscle group collection	FCR, FDS, etc. Auxiliary extorsion wrist	FDS, FCU, etc. Antagonistic flexor collection

#### Synergetic similarity among different movements

To explore the similarities of the muscle activation patterns among different upper extremity movements, we used Spearman’s correlation analysis to compare the average muscle synergy matrix. Table 3 shows the results of the correlation coefficient *r*, significance *p*, and *t*-test statistics *t* with a significance level of 5%. The results showed that there was a highly significant similarity in the HO-WE, HO-SU, and WE-SU groups (*p* < 0.001), a significant similarity in the HO-WF, HO-PR, WF-PR, and WF-WE (*p* < 0.05), and no significances in other groups (*p* > 0.05).

**TABLE 3:** Spearman’s correlation of representative muscle synergies matrixes during different movements.

Synergies	HO-HC	HO-WF	HO-WE	HO-SU	HO-PR	HC-WF	HC-WE	HC-SU	HC-PR	WF-WE	WF-SU	WF-PR	WE-SU	WE-PR	SU-PR
*r*	0.090	0.549	0.620	0.634	0.380	0.128	0.330	0.206	0.265	0.376	0.286	0.537	0.579	0.312	0.169
*p*	0.6232	0.0012	0.0002	0.0001	0.0317	0.4837	0.0645	0.2573	0.1424	0.0339	0.1130	0.0015	0.0005	0.0825	0.3565
*t*	0.4964	4.3287	3.5936	3.4869	2.2530	0.7091	1.9195	1.1547	1.5066	2.2232	1.6326	4.4880	3.8988	1.7966	0.9365

### Shared synergy and special synergy

To further analyze the similarity of the muscle activation patterns among various movements, we used Spearman’s correlation analysis on each module to explore the shared synergy and special synergy. Table 4 shows the correlation coefficient *r* and the significance *p*-value of each synergetic module between different movements. We found that the correlation coefficient value of the $\bar{W_{3}}$ module was basically greater than 0.500, indicating that all movements shared the module $\bar{W_{3}}$ composed of BB. Although there yielded a low linear correlation of the $\bar{W_{3}}$ module between WF-PR and SU-PR groups, they still had similarities. For example, the module $\bar{W_{4}}$ in the HO-WE group had a correlation coefficient of 0.570 and a 95% significant correlation, while modules $\bar{W_{1}}$ and $\bar{W_{3}}$ were 99% significantly correlated with a strong correlation. According to the description in Section “2.6 Statistical analysis,” the modules, $\bar{W_{1}}$, $\bar{W_{3}}$, and $\bar{W_{4}}$ were the shared synergy for the HO-WE group. Additionally, we found a significantly high similarity in $\bar{W_{3}}$ module among all six types of movements, especially for HO-WE, HO-SU, and WE-SU groups, while multiple groups of movements had no statistical similarity.

**TABLE 4:** Spearman’s correlation coefficients among muscle synergetic modules from different movements.

Synergyvector	$\bar{W_{1}}$	$\bar{W_{2}}$	$\bar{W_{3}}$	$\bar{W_{4}}$
*HO*−*HC*	0.062	0.002	0.662**	0.047
*HO*−*WF*	0.405	0.302	0.684**	0.298
*HO*−*WE*	0.783**	0.004	0.799**	0.570*
*HO*−*SU*	0.807**	0.067	0.702**	0.569*
*HO*−*PR*	0.173	0.009	0.685**	0.194
*HC*−*WF*	0.049	0.549*	0.631**	0.005
*HC*−*WE*	0.012	0.134	0.735**	0.224
*HC*−*SU*	0.081	0.015	0.684**	0.235
*HC*−*PR*	0.054	0.470	0.644**	0.001
*WF*−*WE*	0.263	0.024	0.885**	0.021
*WF*−*SU*	0.316	0.134	0.820**	0.093
*WF*−*PR*	0.399	0.589*	0.483	0.869**
*WE*−*SU*	0.877**	0.340	0.790**	0.525*
*WE*−*PR*	0.021	0.001	0.766**	0.006
*SU*−*PR*	0.066	0.019	0.412	0.065

*_P_ < 0.05, **_P_ < 0.001.

**FIGURE 5:**
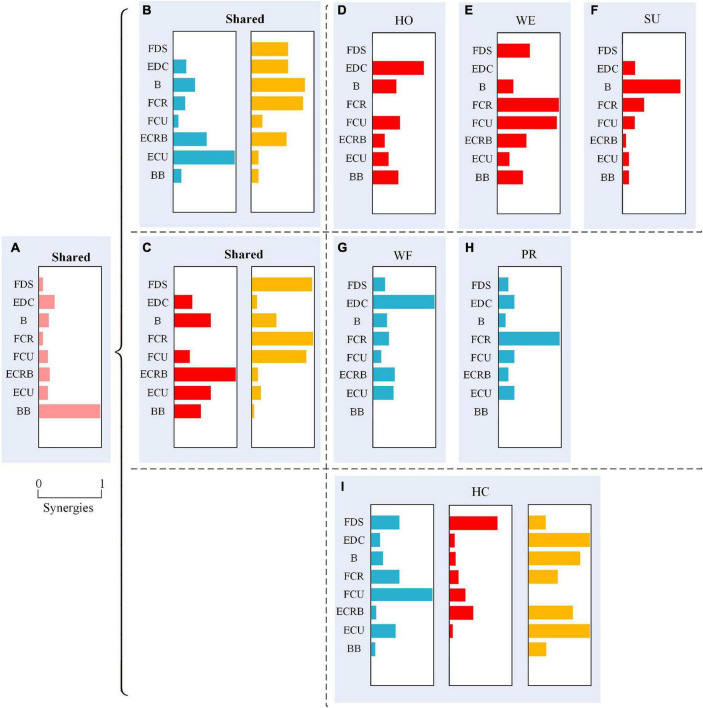
A mixture of synergies shared across different movements and synergies for specific movements showing six movements have a shared synergetic module **(A)**; hand open (HO), wrist extension (WE), and supination (SU) have two shared synergetic modules **(B)**; wrist flexion (WF) and pronation (PR) have two shared synergetic modules **(C)**; and the special synergetic modules **(D–I)** of six movements.

To provide an intuitive understanding of the shared synergy and special synergy of all upper extremity movements, we presented them in a mixture of synergies as described in Figure 5. Figure 5A shows the shared synergetic module $\bar{W_{3}}$ largely of BB, which was the shared synergy of six types of movements. Additionally, Figure 5B shows the shared synergetic modules, $\bar{W_{1}}$ largely of extensor muscles and $\bar{W_{4}}$ largely of flexor muscles, for HO, WE, and SU movements, and Figures 5D–F show the special synergetic module $\bar{W_{2}}$ for HO, WE, and SU movements, respectively. The special synergy for HO was a synergetic module based on the active muscle EDC, for WE was based on the antagonistic muscles FCR and FCU, while for SU, it was one of the active muscles B. Figure 5C also shows the shared synergetic modules $\bar{W_{2}}$ and $\bar{W_{4}}$ for WF and PR movements, and Figures 5G, H show the special synergetic module $\bar{W_{1}}$ for WF and PR movements, respectively. The special synergy for WF consisted largely of the antagonistic muscle EDC, while for PR, it consisted largely of one of the active muscles FCR. Compared with other movements, the HC movement only shared the module $\bar{W_{3}}$ and it’s special synergy were shown in the Figure 5I.

## Discussion

This study illustrated a relatively similar and stable synergy structure under a movement in humans. The activated muscles are combined into different modules according to their functions, which verifies the hypothesis of the modular control with hierarchy in the CNS (Cheung et al., 2009a; Hagio and Kouzaki, 2014; Santello and Lang, 2014). Our study showed that muscle synergy was effective for CNS in solving the problem of complex movement control with multi-degree of freedom which can produce different movements. Similar results are also found in the research on potential movement behavior (Roh et al., 2011). The synergetic effect gathers related muscles and simplifies the control of special biomechanical characteristics by activating muscle groups together, such as angle and direction. It also provides a transition for movement control from task-level goals to executive-level commands (Safavynia and Ting, 2013; Min et al., 2018, 2020).

Many studies have confirmed that the low-dimensional structure of muscle synergy can express the electrical signal generated by the specific spatiotemporal combination (Cheung et al., 2009b; Russo et al., 2014). For example, Filipe Barroso et al. (2013) found that the EMG signal under different movements was reconstructed into a series of spatial structure combinations, which can quantitatively analyze the muscle activation. Our results about the trend of activation coefficients also reflect that the corresponding modules of modulation activation have similar relationships in different individuals, with the CNS as the control center of human movement production modulation. Furthermore, the activation coefficient changes with time, which reflects the changing excitation contribution of each module to the muscle synergy mode (Geyer and Herr, 2010; Balasukumaran et al., 2020). However, the relationship between the activation scale and the neural control remains to be further studied.

In this study, we determined the synergetic module as 4 under the six types of upper extremity movements and believed that the number of synergetic modules was sufficient to explain the changes in the EMG signal recorded during movement. As Figure 2 shows, under synergy number 3, the synergy matrix *W* had the BB with more components only in *W*_3_, which meant that the BB muscle cooperated with other muscles to maintain the stability of the upper limb, and there was no obvious contribution difference between the muscle components in other synergy matrix component, so the relationship of specific synergy could not be clarified. However, the contribution of each muscle component in the synergy matrix was obviously different in the condition of synergetic number four, which could show the different synergetic relationships in the WF movement. In addition, the changes in the activation scale coefficient *C* with time showed that each synergetic module was modulated by the corresponding activation scale coefficient at different times, and the activation level of different synergetic modules also changed accordingly, which reflects the excitation contribution rate *C* to muscle activation pattern. A similar conclusion is also found in the study on the collaborative extraction of human walking and cycling muscles (Barroso et al., 2013), while in the muscle synergy research on frogs swimming, jumping, kicking, and other actions, five synergetic modules are extracted to reconstruct EMG signals (Roh et al., 2011). Hence, we can infer that the modular number of muscle synergy is fewer in some low-complexity movements, while in some high-complexity movements, more modules may be called by CNS to control movement which can ensure the correct execution of the task.

Our results also showed that the upper extremity movements possessed both shared synergetic modules with higher similarity and special synergetic modules without statistical similarity. The module $\bar{W_{3}}$ under the HC is shared by the other five movements, which is consistent with the conclusion that there may be shared synergistic effects in multiple behavioral activities in related studies (Barroso et al., 2015). Meanwhile, we also pointed out the special synergetic modules by comparing the correlation coefficients. For example, the special synergetic module is their own module $\bar{W_{2}}$ for the movement of HO, WE, and SU. Thus, we infer that the CNS can activate shared modules such as $\bar{W_{1}}$ or $\bar{W_{3}}$ and select an appropriate subset such as $\bar{W_{2}}$ to form a muscle synergetic pattern when controlling certain upper arm movements.

Finally, we found that the changing trend of the scale coefficient with time was obviously different by comparing the shared and special synergetic modules under different movements. When performing a motor task, the CNS controls the muscles to form different movements by selecting a certain number of synergies with the modulation change of the synergetic module, the excitation contribution of each muscle, and the activation scale coefficient. The synergistic effect in humans is also in line with a relatively primitive solution of the nervous system of spinal animals such as frogs (Roh et al., 2011). The nervous system uses the direct inhibition or activation of muscle synergy to adaptively express more precise behaviors by breaking or avoiding the synergy structure, that is, the process of synergy modulation.

The limitation of this study is that it only explored muscle activation and synergism in the time domain, but not in the frequency domain. This may lack the exploration of multi-level conclusions. In future studies, we will add the frequency domain to see the muscle synergy in different frequency bands. By doing so, a conclusion can be made whether the muscle synergetic pattern fits well with the time domain. Another interesting aspect would be studying the setting of the thresholds for r, which can measure the shared synergy. Since the selection of the *r* threshold is very important for shared synergy, we will consider updating the algorithm in the future study, so that *r* can be selected adaptively for different tasks and individuals. Additionally, we only explored the muscle activation patterns in healthy humans, while less referred to the patients with motor dysfunction, such as stroke. For patients, the activation and inhibition of muscle synergy may be relatively weakened, so the muscles contract and expand chaotically due to the synergistic effect, resulting in a joint response of motor control. Therefore, the exploration of muscle synergy provides a more theoretical basis for clinical application.

## Conclusion

This study focused on the muscle activation patterns and the similarity of muscle synergy under different movements. We found that the synergetic pattern among subjects was highly similar in the same movement, which showed that the muscle synergetic module called by the central nervous system was task oriented. Additionally, this study indicated the conclusion that muscle synergy between three groups of tasks, namely, HO-WE, HO-SU, and WE-SU, showed a significant similarity, which meant that there were shared synergies among certain movements. Furthermore, this result confirmed the shared synergy and special synergy among different movements. The special synergetic modules meant some unique modules without participating in other movements. In summary, CNS forms different movements by selecting synergetic modules, with the changing of modulation mode of synergy, excitation contribution of each muscle, and the activation scale coefficient. This article can effectively describe the muscle activation pattern under different movements and provide a basis for exploring the activation pattern of motor control. Future studies will include an exploration of the multi-level synergy features in the frequency domain and the adaptability of conclusion in other parts of the body, such as elbow flexion and extension. Furthermore, we will investigate whether the same conclusions exist in patients with motor dysfunction, such as patients with stroke.

## Data availability statement

The datasets presented in this article are not readily available because of privacy and ethical restrictions. Requests to access the datasets should be directed to XC, xlchen@ysu.edu.cn.

## Ethics statement

The studies involving human participants were reviewed and approved by Ethical Review Board of Yanshan University. The patients/participants provided their written informed consent to participate in this study.

## Author contributions

XD, YF, and JY collected the data. XC, XD, and YS analyzed the data and drafted the manuscript. XC, YJ, and XL were responsible for reviewing relevant literature. XC, LZ, PH, and PX revised and determined the final manuscript. All authors contributed to the article and approved the submitted version.
